# Imagination vs. routines: festive time, weekly time, and the predictive brain

**DOI:** 10.3389/fnhum.2024.1357354

**Published:** 2024-04-26

**Authors:** Alessandro Bortolotti, Alice Conti, Angelo Romagnoli, Pier Luigi Sacco

**Affiliations:** ^1^Department of Neuroscience, Imaging, and Clinical Sciences, University “G. D'Annunzio” of Chieti-Pescara, Chieti, Italy; ^2^School of Arts, University of Kent, Canterbury, United Kingdom; ^3^metaLAB (at) Harvard, Cambridge, MA, United States

**Keywords:** predictive coding, active inference, festive time, weekly time, arts, culture

## Abstract

This paper examines the relationship between societal structures shaped by traditions, norms, laws, and customs, and creative expressions in arts and media through the lens of the predictive coding framework in cognitive science. The article proposes that both dimensions of culture can be viewed as adaptations designed to enhance and train the brain’s predictive abilities in the social domain. Traditions, norms, laws, and customs foster shared predictions and expectations among individuals, thereby reducing uncertainty in social environments. On the other hand, arts and media expose us to simulated experiences that explore alternative social realities, allowing the predictive machinery of the brain to hone its skills through exposure to a wider array of potentially relevant social circumstances and scenarios. We first review key principles of predictive coding and active inference, and then explore the rationale of cultural traditions and artistic culture in this perspective. Finally, we draw parallels between institutionalized normative habits that stabilize social worlds and creative and imaginative acts that temporarily subvert established conventions to inject variability.

## 1 Introduction

Human culture manifests itself in two intertwined forms: societal structures shaped by traditions, norms, laws, and customs, and the creative expressions in arts and media that are products of our individual and collective imagination (Alesina and Giuliano, 2015; Shao et al., 2019; Brown, 2021). Scholars have long contemplated the intricate relationship between these two cultural spheres and their respective functions. In this paper, we examine such relationship through the lens of the predictive coding framework in cognitive science (Huang and Rao, 2011). Predictive coding suggests that our brains are essentially prediction machines, continuously creating and updating internal models of the world to anticipate future states and minimize surprise (Shipp, 2016; Pezzulo et al., 2022). We propose that both dimensions of culture can be viewed as adaptations designed to enhance and train the brain’s predictive abilities in the social domain. The former disseminates models tested by groups to facilitate social coordination (Fallucchi and Nosenzo, 2022), while the latter offers simulated environments to challenge assumptions and broaden conceptual possibilities (Pezzulo et al., 2021). Traditions, norms, laws, and customs, and their ideological encapsulations (Wheeler et al., 2020), foster shared predictions and expectations among individuals, thereby reducing uncertainty in social environments. Compliance with these elements signals a commitment to the community and facilitates mutual inference of others’ probable behaviors and reactions irrespectively of their unobservable mental states (Uttich and Lombrozo, 2010). Conformity to conventions also aids in coordinating collective goals (Centola and Baronchelli, 2015). Research in social learning illustrates how the cultural transmission of norms leverages wisdom accumulated over generations to equip members with useful predictive frameworks (Bisin and Verdier, 2023).

In contrast, the arts and media expose us to simulated experiences that explore alternate social realities, venturing far beyond familiar life circumstances (Boyd, 2009). Fiction, music, visual arts, among others, provide spaces for cognitive play to rehearse counterfactual simulations, test possibilities from different perspectives, and open new trajectories for thought (Dancygier, 2011, p. 35). This allows the predictive machinery of the brain to refine its capacities through exposure to a wider array of social situations and scenarios (Oatley, 2016). While traditions conserve successful patterns, the arts disrupt familiar conventions to actively push conceptual boundaries (Galenson, 2008). Despite their remarkable differences, we argue that both cultural forms serve complementary functions in honing the brain’s ability to predict and adaptively navigate the social world. By integrating predictive coding theory with existing interdisciplinary research on culture, arts, and media, we put forth a new perspective on culture as an adaptive system shaped by the imperative of the predictive social brain.

The paper is structured as follows. We first overview key principles of predictive coding and active inference that provide a framework for addressing prediction-driven learning and behavior. We then explore the respective functions of cultural traditions and artistic culture in reducing uncertainty through the propagation of useful social predictive models versus expansive exploration of alternatives. Core hypotheses are that traditions minimize collective surprise by stabilizing and transmitting community-tested models, whereas the arts facilitate the development of flexible generative capacities and social cognition.

By tracing parallels between institutionalized normative habits that stabilize social worlds and creative and imaginative acts that temporarily subvert conventions to inject variability, we point at culture as an essential resource for human survival and effective navigation of physical and social environments (Dissanayake, 2015). Evidence from history and social science reveals how the interplay between conservative and disruptive cultural forces drives artistic innovation, technological creativity, and paradigm shifts (Sassoon, 2006). We conclude by proposing adaptive roles for the dual cultural heritage of compressed traditions and imaginative simulated worlds in training the predictive capacities that make humans such a cognitively unique species.

## 2 The predictive coding framework

Over the past two decades, predictive coding has emerged as a powerful model for understanding perception, cognition, and brain function (Rao and Ballard, 1999; Clark, 2013). At its core, this model posits that the brain is in a constant state of generating top-down perceptual hypotheses or predictions based on prior experience. These predictions are then matched against actual sensory data. Perception, therefore, is not a passive process but an active one. It involves confirming or revising internal predictive models to best explain the external causes of sensory inputs (Friston et al., 2016). This perspective suggests that our brains are essentially prediction machines, continuously creating and updating internal models of the world to anticipate future states and minimize surprise. This framework has been exceptionally successful at explaining low-level perceptual abilities and the neural processes that underlie them. It has also been claimed that predictive coding can account for higher-level cognitive processes like theory of mind, mirror neurons, emotions, aesthetics, self-awareness, consciousness, and disorders of cognitive function such as schizophrenia and autism (Kilner et al., 2007; Spratling, 2008a,b, 2016a; Koster-Hale and Saxe, 2013; Van de Cruys et al., 2022). In essence, predictive coding provides a unified theory of brain function that spans from perception to cognition. In particular, it offers a comprehensive framework for explaining perception, cognition, and action in terms of fundamental theoretical principles and neurocognitive architectures (Hohwy, 2020; Parr et al., 2022).

In its classical formulation, predictive coding provides a specific Bayesian framework for understanding the perpetual interactions between top-down predictions and bottom-up sensations in shaping perception and guiding learning (Friston, 2005).

Prediction errors, which denote the disparities between anticipated outcomes and actual observations, play a pivotal role in propelling the refinement and adaptation of internal models, ultimately enhancing the accuracy of future predictions. This iterative process of aligning predictive models with real-world data to minimize discrepancies closely parallels the principles of Bayesian inference. This adaptive mechanism empowers the brain to fine-tune its probabilistic representations of the underlying causal framework within the environment (Friston and Kiebel, 2009a,b). This process is a fundamental aspect of learning and cognition, allowing us to continuously update our understanding of the world based on new information. In essence, our brains are constantly learning from prediction errors, adjusting our mental models and expectations to better match the reality we experience. This ongoing process of learning and adaptation is what allows us to navigate our complex and ever-changing environment with remarkable efficiency and accuracy.

The foundational tenets of predictive coding align harmoniously with Friston’s free energy principle, which posits that biological systems are driven to diminish surprise by minimizing discrepancies between their predictions regarding sensory inputs and their internal states (Friston et al., 2006; Friston, 2010). Rather than functioning as a passive recorder of sensory information, the brain serves as an active inferential engine, continually striving to anticipate the sensory outcomes arising from the external factors influencing its inputs. In this light, perception is more akin to scientific hypothesis testing than to a mere collection of sensory experiences (Gregory, 1980), with all its related, peculiar cognitive attitudes such as, for instance, the stubbornness in sticking to certain hypotheses even in the face of contradictory evidence (Yon et al., 2019).

Support for the predictive coding framework has grown exponentially across neuroscience domains in the last decades. Predictive signals have been identified across cortical hierarchies and sensory modalities, from early visual pathways to higher-order multimodal association areas (Mumford, 1992; Summerfield et al., 2006; Bastos et al., 2012). For instance, predictive response suppression occurs when expected stimuli evoke a muted response relative to unexpected inputs (Summerfield and de Lange, 2014). This is consistent with the model where top-down predictions are subtracted from or compared against bottom-up signals, minimizing prediction errors for anticipated inputs (Friston, 2005). The functional organization of sensory pathways also aligns with the hierarchical message-passing structure proposed by predictive coding, where backward connections convey predictions and forward connections transmit prediction errors up the cortical hierarchy (Friston, 2008; Shipp, 2016). Dynamic causal modeling of brain imaging data provides further evidence that bottom-up and top-down connections interact asymmetrically in a manner fitting predictive coding principles (Friston, 2016). Moving from such early foundational results, the recent literature has confirmed the robustness and broad applicability of the predictive coding framework in a variety of domains (Nave et al., 2020).

Computational models suggest that predictive coding mechanisms perform well on perceptual tasks like visual object recognition and language processing (Uran et al., 2022; Caucheteux et al., 2023). Machine learning algorithms based on predictive coding architectures can perform robust pattern recognition, particularly in noisy environments or with incomplete inputs (Salvatori et al., 2022; Straka et al., 2023). This demonstrates the potential of leveraging generative models of probable causes rather than just sensory data.

Beyond perception, predictive coding theory has expanded to address how the brain predicts complex dynamics across multiple timescales, including not just sensory signals but also external events, internal states, and abstract rules or relationships (Kiebel et al., 2009; Bubic et al., 2010). Humans deploy an integrated system of multilevel predictive models spanning motor control, interoception, motivation, memory, theory of mind, value learning, and conceptual knowledge (Clark, 2013; Pezzulo et al., 2015).

Active inference extends predictive coding from passive model updating into how organisms take actions to seek out experiences expected to reduce overall uncertainty in their predictive models, not just immediately but over the future (Friston et al., 2009, 2017a). This provides a foundation for addressing prediction in decision-making in ecological settings.

Predictive coding has thus emerged as a reference model for rethinking cognitive function through a unifying Bayesian lens. It provides a framework for explaining numerous perceptual, behavioral, and neural response patterns as reflecting the imperative of a prediction-driven learning system. Its unique strength lies in capturing the tight coupling between top-down and bottom-up processes which leverage prior knowledge to parse the onslaught of sensory data. By postulating concrete mechanisms for learning as model optimization through prediction error correction, predictive coding moves from abstract metaphors of the mind as an inference machine toward formalizable computational models with increasing explanatory and integrative power. Grounded in Bayesian probability and information theory, predictive coding aligns with the increasingly influential view of the brain as a near-optimal probability estimation machine. In other words, predictive coding serves as a bridge connecting abstract theoretical concepts to empirical observations, offering a versatile framework that enhances our understanding of cognitive processes across various subfields of neuroscience. The integration of Bayesian principles into the predictive coding model (Spratling, 2016b) emphasizes the brain’s inherent ability to engage in near-optimal probabilistic estimations, further cementing its position as a valuable cornerstone in the study of human cognition and perception.

In the sections that follow, we apply active inference and predictive coding to provide a novel perspective on the complementary evolutionary functions of the two major forms of human culture.

## 3 The complementary functions of culture: play, practice, and cultural evolution

Culture in its dual instantiations fulfills complementary learning needs of the predictive brain. Norms and traditions conserve successful predictive patterns and transmit wisdom that has proven adaptive to the local niche over time while reducing uncertainty in social environments through shared conventions. Artistic culture provides more flexible simulation spaces to actively push beyond existing patterns and entrenched ideas to explore new perspectives and models.

The interplay of conserving social practices and imaginative exploration parallels the rhythms of weekly routine and festive time observed in many societies across history. Durkheim (1915) noted that festivals mark a break from mundane workday life focused on instrumental activities. Artistic events open up spaces devoted to the exploration of imaginative worlds, where social norms relax, and role play expands beyond habitual identities in a way not sanctioned during routine weekly time. Likewise, in the thinking of William James one finds a contraposition between the ‘epiphanic’ sphere of creative imagination and the ‘average’ sphere of mundanity (Pawelski, 2007). Again, Bakhtin’s (1984) notion of carnival as a temporary suspension of the ordinary rules and hierarchies of socioeconomic and political organization and as attainment of a new communitarian awareness through sensual and bodily engagement is another important perspective into the institutionalized structuring of festive time as a sociobiological necessity.

But the suspension of the rules of weekly time need not happen in circumscribed, festive time slots only. In this regard, it is important to consider Shklovsky’s (2007) notion of ‘defamiliarization’, namely the artful representation of familiar objects and situations as if seen for the first time. Through defamiliarization, even the elements of everyday reality can be turned into doors toward imaginary worlds and narrative experimentation. Shklovsky (2007) illustrates at length how this technique is widely used in poetry and literature to de-structure the mechanisms of perception to pave the way to different, more profound forms of experience, suggesting that the arts can carve ‘epiphanic’ bubbles also within the frame of the most mundane aspects of existence. Gamoneda (2020) builds upon Shklovsky’s (2007) approach to analyze how defamiliarization may be intended as a form of cognitive estrangement with important neurocognitive implications in terms of embodied simulation and reframing of sensory and emotional experiences.

Turner (1969) expanded on these ideas with his concept of “liminality” – transitory spaces that break social norms and allow free experimentation. As for Bakhtin (1984), the carnivalesque atmosphere of festivals permits temporary inversion of hierarchies, parody of leaders, and exploration of taboo ideas that would normally be off-limits. We can interpret this ebb and flow between tradition-bound routine and creative festive periods as giving predictive brains space to cycle between preserving useful patterns and expanding into new possibilities.

Sutton-Smith (2009) proposed that the human appetite for generating varied imaginative worlds through games, art, and make-believe serves an adaptive cognitive function he termed “the ambiguity of play.” The arts and games allow safe spaces to manipulate meanings, construct virtual realities, and explore identities in deliberately ambiguous ways disassociated from worldly consequences. By engaging with hypothetical scenarios on both personal and social scales, thinking through liminality facilitates cognitive development and mental flexibility and prepares us for unpredictable change (Thomassen, 2015, p. 40–1).

Building on these insights, we can understand festive time as a crucial opportunity for developing individual skills and social ideas that overcome the constraints of tightly regulated weekly time (Ehrenreich, 2007). The loosening of social control mechanisms during festivity allows not just frivolous escapism but serious collective experimentation with alternative models and meanings (Páez et al., 2015). Artistic culture provides low-cost simulated sandboxes ideal for this exploratory predictive work (Boal, 2005). Many historical examples illustrate the interplay between conserving traditions and visionary imagination stimulating cultural evolution. Classical Greek theatre performances explored taboo themes like incest and patricide that defied traditional mores but did so in highly ritualized contexts that were demarcated from real life. Hybridizing fanciful theatrical simulations and reasoned debate in the political sphere catalyzed pioneering advances in democracy, rhetoric, law, and philosophy that deeply shaped later cultures (Meineck, 2017). Meineck draws upon theories of ancient Greek theater and ancient rituals to elaborate on the concept of liminal spaces. In this context, liminal spaces represent locations where social norms can be momentarily suspended or reversed (McKenzie, 2004). This suspension of social norms allows people to experience a sense of liberation and challenge social conventions (Seale-Collazo, 2012). Liminal spaces thus provide opportunities for individuals to expose themselves to unconventional situations, opening the door to new perspectives and knowledge (Pielichaty, 2015). In this sense, liminal spaces offer learning opportunities and contribute to cognitive development through the exploration of surprising social dynamics and the acquisition of a broader understanding of the world from unexpected angles.

The European Renaissance provides another example where tensions between conservative and imaginative cultural elements spurred paradigm shifts. As royal patrons began supporting innovative secular painting, sculpture, and architecture rather than solely religious works, exposure to these novel perspectives expanded audiences’ perceptual predictions and worldviews and radically transformed the notion of creative agency (Heller, 1978). The humanistic emphasis kindled by the arts stimulated scientific creativity and likely coevolved with more pluralistic social values (Greenblatt, 2019).

Throughout history, the intermingling of traditional habits and artistic innovation has catalyzed advances in fields as varied as music, visual arts, literature, and even mathematics and science (Root-Bernstein, 2003; Charyton, 2015). Playful challenges to orthodoxy expand the realm of conceivable models, which can lead to revolutionary syntheses and broader paradigm shifts when new syntheses are adopted by societies (Kuhn, 1962). Sperber (1996) synthesized these perspectives in his epidemiological theory of how representations spread through cultures. He argued that the tensions between predictable traditions and unfettered artistic innovations produce an endless supply of new mental representations competing for adoption. The most cognitively captivating meanings catch on, spreading through networks via inherent human drives for social learning and memorability. Periodic injections of imaginative variation therefore prevent stagnation and spark new cycles of social creation of meaning.

Adopting the predictive coding theory as a conceptual backbone allows the reframing of this interplay between conservative and disruptive cultural forces as serving essential cognitive ecosystem functions. This cultural cycle of conservatism and innovation parallels the scientific process (Kuhn, 1962). Existing paradigms conserve frameworks honed to explain ordinary cases, while outliers spark paradigm shifts (Sterman and Wittenberg, 1999). Predictive coding proposes that the brain shares this intrinsic drive for theoretical refinement – conserving useful models while continuously probing at the edges to uncover anomalies that necessitate expanding the model repertoire to reduce surprise.

This evolutionary interplay between traditions and disruptive art may have been critical for the cumulative generative capacities that make human culture unique. Donald (1991) proposed that the dual heritage of ritual imitation and mimetic imagination drove our species’ unrivaled cognitive fluidity. The arts may play the essential role of introducing variation and challenging assumptions necessary for cultural evolution (Gabora, 2017), while social norms conserve building blocks between innovations.

Culture therefore appears finely tuned to serve both conservative and disruptive/exploratory predictive drives. Ritualized traditions and norms preserve compressed generative models specialized for coordinating social exchange and minimizing uncertainty. Meanwhile, festive periods and the arts facilitate exploratory simulations and competition between alternative models and worldviews. This dual cultural ecosystem provides a training ground for optimizing the predictive capacities of our brains in both routine and novel contexts, supporting human adaptability and cumulative knowledge growth.

## 4 The predictive function of cultural norms

Cultural norms – the informal rules guiding behavior in groups – provide key social coordinates that structure interactions into predictable patterns. Norms provide members with shared expectations about how others are likely to act in recurrent situations, allowing individuals to anticipate behavior and outcomes (Hawkins et al., 2019). The predictive functions of cultural norms can be linked to René Girard’s mimetic theory. Cultural norms are considered models of acceptable behavior and guide people’s actions through mutual imitation. Mimetic theory suggests that imitation can lead to rivalry and conflicts, but cultural norms serve as a tool to prevent violence, regulating behavior and providing shared rules for resolving conflicts nonviolently. In this context, cultural norms play a key role in creating predictions of behavior within a society (Girard, 2005).

From a predictive coding perspective, cultural transmission of norms trains individuals to develop more accurate generative models about social cause-effect patterns. This process minimizes surprise and enables coordination within a society (Leung, 2015). The transmission of norms involves the selection and socialization of cultural ideas from one generation to the next. Parents play a crucial role in this process, as they strategically choose which cultural ideas to transmit based on their own orientations and perceptions of what is normatively important in their culture (Silverstein and Conroy, 2008). The perceived norms perspective suggests that parents reference these norms to guide their actions and goals (Kastel et al., 2023). Through cultural transmission, individuals learn and internalize these norms, which shape their behavior and decision-making processes. The cultural transmission of norms contributes to the development of accurate generative models that help individuals navigate social interactions and coordinate effectively within their cultural context.

### 4.1 Conceptualizing social norms

Philosophers and social scientists have long recognized that informal social norms and conventions play a central role in human culture, that is distinct from that of formal laws. David Hume emphasized how conventions enable complex cooperation between individuals with limited altruism (Bruni and Sugden, 2000). Émile Durkheim viewed norms as collective representations binding society together (Durkheim, 1964; Gilleard, 2018). Max Weber delineated legal-rational prescriptiveness from traditional authority embodied in customary norms (Spencer, 1970). Bicchieri (2006) synthesized a useful schema for dissecting the components of social norms. She distinguishes between empirical expectations – beliefs about what others normally do in a situation, and normative expectations – beliefs about what others think ought to be done. Shared expectations on both dimensions provide social proof enabling inferences about appropriate behavior (Cialdini et al., 1990; Friston et al., 2011). The violation of norms exposes to social sanctions aimed at stigmatizing unjustified deviance.

This dual structure parallels predictive coding notions of perceptual hypotheses and prior beliefs. Empirical expectations represent compressed accumulated observations about typical behaviors, while normative expectations are more abstract schematic knowledge about permissible actions – similarly to prototypes and causal models. Together, they comprise a social grammar predicting likely events and reactions during recurrent situations, of which we start to understand the neurobiological foundations (Duerler et al., 2022).

### 4.2 Psychological drivers of norm compliance

A wealth of research highlights strong psychological drivers behind norm compliance and enforcement. Classical conformity studies demonstrate that individuals alter opinions and behavior to match group majorities, particularly when uncertain or stressed (Asch, 1956; Zhang et al., 2016). Obedience experiments reveal a willingness to obey authority figures even when directives violate personal morals (Milgram, 1974; Haslam et al., 2014). Enforcement studies show a willingness to punish norm violators, even at personal cost and even for norms recently introduced in laboratory settings (Fehr and Fischbacher, 2004). What mechanisms underlie these striking conformity patterns? Informational influence suggests looking at others for evidence about reality, especially in ambiguous situations. Normative influence describes compliance to avoid ridicule or gain approval. Both serve uncertainty reduction functions complementing predictive coding notions (Deutsch and Gerard, 1955). Uncertainty Management Theories argue how group identification, conformity, and schema reliance all aid in managing an unpredictable social world (Hogg and Adelman, 2013). Shared norms provide expectational templates guiding the perception and interpretation of ambiguous social stimuli (Chang and Koban, 2013). Deviating is at risk of sanctions and unclear reactions, whereas adherence demonstrates commitment, reducing uncertainty about one’s standing (Mullin and Hogg, 1998).

Computational models demonstrate how norms can arise from simple interaction rules. Agent-based models show the emergence of stable cooperation in social dilemmas when agents adopt strategies to meet group expectations (Axelrod, 1997; Macy and Flache, 2002; Hanaki et al., 2007). In Bayesian models, agents infer hidden group conventions from sparse observations of others’ behavior (Shafto et al., 2014). These dynamics converge on stable equilibrium normative behavior without global knowledge or top-down enforcement. Such models provide a framework for understanding the evolution of societal norms and their impact on individual and group behavior. They offer insights into how norms can change over time and how they can be influenced by external factors such as cultural shifts or technological advancements.

Together, these psychological and computational mechanisms highlight how internalized norms reduce uncertainty by training expectations about others’ behaviors and likely consequences of potential actions. This enables a fluent navigation of the fast-paced social world. These mechanisms underscore the importance of norms in shaping our social interactions and our understanding of the world around us. They emphasize the role of norms in guiding our actions and decisions, ultimately influencing the trajectory of our social lives.

### 4.3 Predictive functions of cultural learning

Traditions passed across generations embody accumulated knowledge about recurrent situations and preparations for likely future scenarios (Boyd and Richerson, 1985). Rituals, including extreme ones (Fischer and Xygalatas, 2014) provide procedural knowledge about coordinated cultural practices that minimize uncertainty through collective synchronized actions (Watson-Jones and Legare, 2016). Tomasello (2016) proposes that the human capacity for complex cumulative culture rests on abilities to understand shared goals, make recursive inferences about others’ mental states, and learn from pedagogy. This allows comprehension of artifacts and practices as solutions to commonly understood problems facing the group, facilitating cultural model transfer. Traditions and rituals serve as a bridge between the past, present, and future, allowing societies to maintain continuity and coherence. They also foster a sense of identity and belonging among group members, strengthening social bonds, and promoting group cohesion (Jackson et al., 2018). The ability to understand shared goals and infer others’ mental states is crucial for effective communication and collaboration (Tomasello et al., 2005) and has complex neurobiological underpinnings (Saxe, 2006). It enables individuals to anticipate others’ actions, coordinate their own actions accordingly, and work together toward common objectives. This, in turn, enhances the group’s ability to adapt to changing circumstances and overcome challenges. Preston and Wegner (2007) detail how implicit cultural learning mechanisms including imitation, emotional contagion, and norm adoption allow rapid indirect acquisition of vast group knowledge. Children discern societal expectations and conventions without need for explicit coaching (Schmidt and Tomasello, 2012; Rakoczy and Schmidt, 2013). Enactment itself strengthens neural pathways for skills and norms through immersive participation in patterned cultural practices, enabling thinking through other minds and the alleged process of inferring other agents’ behavioral expectations (Veissière et al., 2020).

Repetition further cements predictable routine behaviors, if it is supported by specific attentional cues (Musfeld et al., 2023). Ritual gatherings provide regular synchronized affirmation of norms and values (Rossano, 2012). Cycles of festivals and ceremonies structure the cultural calendar into anticipated occasions renewing group identity and bonds (Malinowski, 1922; Whitehouse and Lanman, 2014). This affirms ingroup predictive models by exploiting their causal opaqueness and their impermeability to individual innovation and change (Legare and Wen, 2014). In general, repetitive and ritualistic behaviors serve to reinforce social norms and expectations, thereby promoting stability and order within the group, but they cannot be manufactured at will (Hobson et al., 2017). They provide a sense of predictability and control, reducing uncertainty and enhancing the group’s collective sense of security (Lang et al., 2022). Moreover, the cyclical nature of festivals and ceremonies not only renews group identity and strengthens social bonds, but also provides a temporal framework that guides social interactions and activities, functioning as a regulatory mechanism (Tonna et al., 2019). This structuring of social life across time contributes to the formation and maintenance of a shared cultural identity, reinforcing the group’s distinctiveness and cohesion.

At the end, the affirmation of ingroup predictive models through these practices underscores the importance of shared understanding and consensus in maintaining social harmony. It highlights the role of collective beliefs and values in guiding individual and group behavior, shaping social dynamics, and influencing the group’s overall trajectory.

### 4.4 Neuroscience of social learning

Neuroimaging studies reveal the possibility of shared neural circuits for learning hierarchical sequences across sensorimotor, linguistic, and social domains. Meta-analyses show moreover that social norms representation and violation are associated with different, specialized brain circuits (Zinchenko and Arsalidou, 2018). Value learning research finds that social norms rapidly acquire motivating valence through dopamine habituation pathways similar to primary rewards, providing intrinsic motivation to conform (Klucharev et al., 2009). fMRI studies show greater striatal responses for choices aligning with perceived social norms (Nook and Zaki, 2015), consistent with predictive coding notions of expected reward and precision (Friston et al., 2014). Together these findings indicate that social norms harness general hierarchical sequence learning abilities while tapping into neurocognitive systems for rewards, mentalizing, and uncertainty-driven learning. This allows an indirect acquisition of complex cultural knowledge. All these findings suggest that the brain’s ability to learn and adhere to social norms is not only a product of social conditioning, but also a fundamental aspect of human neurobiology (Cikara and Van Bavel, 2014). The brain’s reward system, which is crucial for motivation and learning, appears to be intrinsically linked to our ability to understand and follow social norms (Montague and Lohrenz, 2007).

In addition, the use of neuroimaging techniques such as fMRI has provided valuable insights into the neural mechanisms underlying social norm compliance (Spitzer et al., 2007; Bellucci et al., 2018; Toelch et al., 2018). These studies have revealed that abiding by social norms is also associated with activation in brain regions involved in reward processing and decision-making, suggesting that social norms may be encoded in the brain as rewarding behaviors (Ruff and Fehr, 2014).

Moreover, the role of dopamine in social norm compliance suggests a neurochemical basis for our tendency to conform to societal expectations. Dopamine, a neurotransmitter involved in reward and motivation, may play a key role in reinforcing adherence to social norms and promoting social cohesion. These findings highlight the complex interplay between social, cognitive, and neural processes in shaping our behavior and understanding of social norms. They underscore the importance of interdisciplinary research in advancing our understanding of the human social mind (Frith and Frith, 2012).

### 4.5 Predictive coding of social patterns

Predictive coding proposes that the brain’s hierarchical generative models leverage accumulated experience to predict unfolding events, actions, and internal states (Friston, 2005). This framework extends naturally to collective culture. Social norms build upon compressed knowledge about stable patterns in group interactions. Rituals, customs, and practices represent cultural ‘programs’ solving common coordination problems, transmitted across generations. Conformity enforces the reliable propagation of predictively useful models. Ritual gatherings provide regular opportunities to synchronize and entrench model parameters guiding inferences. Predictive coding provides a theoretical framework for understanding how individuals learn and adapt to social norms. It suggests that our brains are constantly updating our beliefs and expectations based on our experiences and observations, allowing us to navigate the social world with greater efficiency and accuracy (Clark, 2013).

Shared culture thus equips individuals with robust prior models for navigating the social world. Compliance demonstrates the reliability of these ‘theories’, minimizing surprise. Violations force an error-driven updating of faulty assumptions. Prosocial behavior and obedience instantiate inferences about others’ mental models based on mature cultural schemas. Furthermore, the concept of shared culture emphasizes the role of collective knowledge and experience in shaping individual behavior. It suggests that our actions and decisions are not solely determined by our personal beliefs and desires but are also influenced by the norms and values of the group to which we belong (Boyd and Richerson, 2005). The notion of compliance as a demonstration of the reliability of cultural ‘theories’ underscores the importance of social validation in reinforcing social norms. When individuals observe others adhering to a particular norm, they are more likely to perceive that norm as valid and beneficial and are thus more likely to adopt it themselves (Cialdini and Goldstein, 2004). The idea of prosocial behavior and obedience as instantiations of inferences about others’ mental models highlights the cognitive processes underlying social interaction. It suggests that our understanding of others’ thoughts and intentions is shaped by our cultural schemas, which provide a framework for interpreting and predicting others’ behaviors (Tomasello, 2014).

In this view, cumulative cultural knowledge acquired through norms provides high-fidelity generative models for fluent predictive processing during social exchanges and collaborative endeavors. While individual learning is slow and costly, leveraging collective wisdom enables rapid indirect acquisition of models attuned to complex cultural patterns over decades or centuries. Common culture therefore functions as a distributed prediction machine minimizing collective surprise.

## 5 Art as playground for the predictive brain

If cultural norms can be seen as encoded predictive patterns, finely tuned to facilitate social interactions, then the realm of artistic culture offers a more pliable domain for the active exploration of novel patterns. Through various mediums such as media, visual arts, literature, music, and performance, humans gain the opportunity to immerse themselves in imaginative worlds, virtual simulations, and playful experiments with identity, having the chance to actively contribute to their unfolding. These experiences extend far beyond the confines of the physical world.

This leads to a compelling question: What intrinsic motivations might drive our brains to invest considerable energy in the generation and interaction with these artificial, imaginary realms? Predictive coding theories hold the key to some intriguing possibilities, as they provide a framework for understanding how the brain predicts, compares sensory data, and adapts to new information. Art, with its variety, complexity, and countless nuances, is an ideal space to explore and apply such theories. It offers a unique opportunity to comprehend how the brain anticipates, interprets, and adjusts to the world through the lens of artistic experiences.

### 5.1 Prediction, play, and possibilities

Predictive coding is not just about reducing immediate prediction mistakes. It’s also about enhancing the adaptability and flexibility of internal models over time, by exploring new ideas and questioning existing assumptions (Schwartenbeck et al., 2013; Friston et al., 2017b). This aligns with the play theory notion that play behavior is impulse-driven learning that is essential for cognitive development (Groos, 1901).

Fictional spaces offer sandboxes where brains can safely toy with alternative simulations unconstrained by risks, resources, or actual outcomes (Clark, 2016; Oatley, 2016). Games provide structured play spaces designed to engage learning drives (Juul, 2011). The arts and media enable counterfactual exploration broadening predictive capabilities beyond local ecological constraints. As already remarked, Sutton-Smith (2009) argued that the human appetite for diverse imaginative worlds serves adaptive functions he termed the “ambiguity of play.” Play loosens anchors to reality, allowing flexible recombination of meanings and concepts. Make-believe provides a play space to manipulate identities and virtual realities while suspending worldly consequences. This facilitates cognitive development and mental flexibility. In this view, arts and fiction allow indulging intrinsic drives to address uncertainty through imaginary exploration, expanding concepts and causal models beyond available experience (Carroll, 1990; Swirski, 2007). Just as children play games to train skills, creative works provide artificial environments to refine and diversify predictive models (Boyd, 2009). Hartung and Willems (2020) propose that fiction and fantasy specifically target gaps in readers’ conceptual networks. The navigation of imaginary worlds thus harnesses the training of generalizable cognitive skills.

### 5.2 Mimetic culture as cognitive playground

Donald (1991) argued that complex mimetic skills enabled humans to construct imagined realities orders of magnitude beyond other species. Ritual and oral myth-telling coevolved with material culture to simulate virtual worlds, constituting a playground for modeling alternate psychic and social realities.

Donald termed this nexus of mimetic cognitive abilities, ritual behavior, oral narrative, and aesthetic culture the “mimetic mind.” He proposed that the generative capacity at the heart of human cognition emerged from the interplay between mimetic imagination and cultural innovation. The arts provided spaces to push mimetic skills, expanding the horizons of possible realities. Across cultures, arts extract deep structural invariances in experience as creative templates (Dissanayake, 2015).

In this view, the universal delight humans find in the arts reflects impulse-driven play behavior that hones cognitive skills by exposing minds to divergent worlds. Aesthetic pleasure may arise from satisfying intrinsic drives to seek out tractable challenges and new possibilities that expand generative capacities (Csikszentmihalyi, 1990). The predictive brain therefore engages with art as a particularly adaptive form of cognitive training.

### 5.3 Cultural learning: the tension between uncertainty reduction and creative imagination

Tomasello (2016) argues that cumulative human culture relies on capacities to discern others’ intentionality and recursively simulate how artifacts solve shared problems. This allows indirect acquisition of models and practices with opaque purposes. Norms and rituals efficiently transfer adaptive cultural knowledge. Boyd and Richerson (1985) detail how cultural learning mechanisms promote the acquisition of group-tested knowledge and behaviors. Younger generations readily adopt cultural patterns, leveraging accumulated wisdom without needing to reinvent solutions from scratch (Dean et al., 2014). Social learning can thus optimize solutions over generations in ways individual learning cannot (Ballinger et al., 2003).

Traditions and norms thereby propagate community-refined predictive models that reduce uncertainty by providing frameworks for addressing many issues that humans recurrently face. Artistic culture complements this conservative function with spaces to challenge assumptions and exploring ulterior possibilities that are instrumental to the evolution of culture (Morriss-Kay, 2010; Kesner, 2014). Tensions between norms and disruptive imaginings stimulate revolutions in perspective that can reshape societies (Poks, 2020). Open-ended simulation abilities allow humans to download culture on vast scales to efficiently equip predictive brains with tools, practices, languages, and bodies of knowledge that are refined and institutionalized across generations through a constant dialectical tension between conservation and disruption (Wheeler, 2003), and that eventually favor the minimization of collective uncertainty (Dissanayake, 2006). The arts may provide essential cognitive sandboxes necessary for evolving culture and discovering new predictive models not easily discerned through individual experience (Wolf, 2020).

### 5.4 Exploration of social worlds

Fiction turns out to be a particularly precious resource devised by humans for the safe exploration of the realm of complex social causality (Fletcher, 2021) that is a characteristic source of uncertainty in human and more generally primate societies (Ramos-Fernandez et al., 2018). Social cognition researchers argue that navigating the tangled dynamics of agency, intention, cooperation, deception, and recursive thinking presents perennial adaptive demands that have left a clear trace in human biological design (Platt et al., 2016; Dunbar, 2018). Shared imaginative worlds provide low-stake training grounds for social competence (Dodell-Feder and Tamir, 2018). The capacity of literary fiction to function as a rich simulation of social worlds has been shown to engage theory of mind reasoning more than popular fiction (Kidd and Castano, 2013, 2019). Reading fiction moreover correlates with performance on social inference tasks (Mar et al., 2006; Mumper and Gerrig, 2017). Transportation into narrative worlds involves inferring characters’ beliefs, motives, and likely behaviors (Fletcher, 2023), and qualitative differences in fiction formats and thematic genres may affect levels of prosociality (Turner and Felisberti, 2018). This “pretense of social interaction” exercises social predictive models by exposing readers to diverse agents and perspectives (Zunshine, 2006), so that fiction provides surrogate experiences and social commentary expanding human intuitive psychology (Oatley, 2016). As the arts simulate alternate social realities as cognitive training, music similarly provides an abstracted relational sandbox. Levitin (2006) argues that musical pleasure stems from tracking nested patterns across timescales and actively predicting upcoming structures. Specific aesthetic manipulations exercise cognitive skills for detecting variation, recursion, and resolution in sequenced patterns, which transfer to social domains (Cross, 2012; Pearce and Rohrmeier, 2012). Generative competition between conventions and creative novelty may drive the cultural evolution of such adaptive learning environments across art forms (Gabora, 2017). The cultural canon can therefore be regarded as a storage of socially validated simulations whose engagement potential relates to their capacity of honing valuable social inference skills.

### 5.5 Neural basis of art and social cognition

Neuroscientific evidence corroborates the existence of links between the arts and social cognition. Both fiction reading and social working memory tasks activate the default network and executive system (Hsu et al., 2015; Tamir et al., 2016). Exposure to predictively stimulating musical patterns recruits neural valuation circuits (Salimpoor et al., 2015), further supporting the idea that humans are intrinsically motivated in seeking novelty in their experiential sphere (Baranes et al., 2014). Such evidence suggests that the arts, and more generally creative, out-of-the-box thinking, activate executive (Beaty et al., 2015) and social inference (van Leeuwen et al., 2022) networks, supporting proposals that engagement with narrative or auditory worlds, among others, provides valuable opportunities for cognitive training. This would also explain why art forms such as music, visual arts and fiction appear intrinsically rewarding, as a result of their value in promoting successful ecological adaptation and not as a pure hedonic stimulus (Lacey et al., 2011; Blain and Sharot, 2021). It also accounts for why brains are intrinsically motivated to devote energy and attention to artificial experiences serving no apparent survival necessity, but superseding attendance of crucial survival functions such as eating and sleeping while deeply immersed in engaging fictional worlds (Perks, 2014). The arts offer a primary experimental space tailored to exercise social cognition capabilities, just as games and play train physical and problem-solving skills, and such goals are heavily prioritized in our biobehavioral design (Kirsch et al., 2016). Culture may then have evolved as a cognitive amplification tool for social animals dependent on recursive mindreading but constrained by risks and costs of unbounded, unregulated real-life exploration.

This perspective integrates theories of play and culture with predictive processing frameworks and research across anthropology, psychology, aesthetics, and neuroscience. It generates intriguing hypotheses about requisite cultural elements for optimizing the adaptive predictive accuracy of human brains across both physical and social domains.

### 5.6 The dynamic toggling between weekly and fesive time: culture as situated cognition

Once understood how weekly and festive time cultures play different but complementary roles, we need to consider what are the socio-cognitive mechaisms that egulate the dynamic toggling between these two spheres. A useful conceptual framework in this respect is culture-as-situated-cognition (CSC) theory (Oyserman, 2016), that highlights the malleability of cultural mindsets and their influence on cognitive processes, judgments, and behavior. Individuals have access to multiple cultural mindsets, such as individualistic, collectivistic, and honor mindsets, which can become temporarily accessible through situational cues. The cognitive effects of these primed mindsets can manifest in various ways, such as influencing attention allocation, reasoning styles, and social judgments (Oyserman et al., 2009; Choi et al., 2016). The interplay between weekly time, characterized by routines and traditions, and festive time, marked by artistic expression and creative exploration, can be understood as a cyclical process that regulates the accessibility of different cultural mindsets. The institutionalization of weekly vs. festive time in terms of the social calendar of weekdays, weekends, and festivities, therefore, acts itself as the social cue that primes the activation of different mindsets.

However, the arts can act themselves as a social priming mechanism, priming temporary mindset shifts also within the fabric of weekly time, as in the already discussed case of Skhlovsky’s defamiliarization techniques. Just as priming tasks in experimental settings can make individualistic or collectivistic mindsets accessible, exposure to artistic experiences can prime cognitive processes associated with individualistic or collectivistic orientations, with the difference that, unlike social cues designed in the laboratory, artistic cues are not only ecological, but also suitably evolved for this purpose. This explains why the arts can become powerful forms of social priming eliciting attitudinal and behavioral change (Klatt et al., 2013; Martin et al., 2018), and why they represent a potentially more promising foundation for behavioral policy interventions than artificial, paternalistic choice architecture ones which struggle to fit the contextual complexity of ecological human environments (Meder et al., 2018). In particular, the priming effects of artistic experiences during festive time are sociobiologically designed to counteract the potential risks of cultural entrenchment and cognitive rigidity associated with prolonged immersion in routine practices. By periodically activating alternative cultural mindsets and exposing individuals to diverse social simulations, festive time fosters cognitive flexibility, adaptability, and the ability to navigate complex social environments.

## 6 The neuroscience of traditions and artistic innovation: the shallow brain hypothesis

Recent advances in cognitive neuroscience lend further support to the conceptual framework proposed here regarding the complementary roles of traditions and artistic innovation in training the predictive capacities of the social brain. In particular, the “shallow brain hypothesis” outlined by Suzuki et al. (2023) provides an intriguing neurobiological perspective that enriches the functional account of culture’s dual nature.

The shallow brain hypothesis questions the dominance of hierarchical models like mainstream versions of predictive coding that assume higher cortical areas to sequentially build up abstract features. However, this line of argument challenges such predicting coding approaches in a way that further corroborates the conceptual foundation of cultural experiences proposed in this paper rather than undermining it. Let us see why. Suzuki et al. (2023) point to extensive evidence that even primary sensory cortices project directly to subcortical areas like the thalamus, basal ganglia, and brainstem. Furthermore, they highlight how all areas, both higher and lower, receive inputs from subcortical structures, constituting a massively parallel architecture. From this shallow neuroanatomical arrangement, Suzuki et al. propose several key computational benefits that may be relevant to navigating cultural environments. First, the direct cortico-subcortical loops allow fast processing and learning in local circuits without sluggish propagation up and down cortical hierarchies. This may align with the proposal that rituals and norms leverage rich sensory-motor links and subcortical habits to quickly entrain generative models, while art provides more unconstrained exploration.

Additionally, the shallow architecture’s convergence in subcortical regions enables flexible selection and combination of diverse cortical inputs. This may lend neurological support to the hypothesized role of subcortical areas in integrating conventions and innovations, also in view of the increasing recognition of the extensive subcortical contribution to cognitive networks (Janacsek et al., 2022; Pessoa, 2024). The basal ganglia and thalamus are positioned to gate and blend outputs from lower and higher cortical processors. The extraordinary computational capacity within local cortical microcircuits also emphasized by the shallow brain hypothesis suggests that both compression of traditions and expansion of innovations might occur within specialized but fully integrated circuits. The generative architectures for constraining versus enhancing conceptual networks may operate in parallel. The shallow brain thesis further elucidates how synaptic plasticity sculpts cortical microcircuits over learning experiences, gradually yielding efficient representations tailored to recurrent situations. This neural tuning account seems to support the idea that norms and rituals cultivate shared generative models optimized for stabilizing social worlds. Finally, Suzuki and colleagues elaborate predictive coding principles showing how brains minimize surprise by matching top-down models against sensory inputs to correct errors. The shallow brain perspective demonstrates how hierarchical and parallel circuits coordinate to implement prediction-driven learning. This neuroscience-grounded predictive processing aligns with and substantiates the overarching framework proposed for cultural forms sculpting social predictive abilities.

The shallow brain hypothesis may be a precious source of research hypotheses to further probe into the conceptual duality presented here between traditions that transmit compressed models and art that expands horizons. Neural evidence confirms cultures harness flexible cortical–subcortical circuits (Nadal, 2013) whose role in the stabilization and adaptation of predictive models attuned to both routine and novel social challenges needs to be further explored. The shallow neuroanatomy of the brain might thus provide a fascinating basis for an elegant conceptual convergence between our current insights about the psychological and biological mechanisms involved in both dimensions of our cultural experience.

This interdisciplinary synthesis of cognitive and brain sciences provides a richer picture of how human predictive capacities intertwine with culture’s dual heritage. Such a conceptual framework appears promising for future research leveraging neuroimaging, neural network modeling, and clinical studies to further elucidate the cognitive and neural processes bridging brains and human cultures. Understanding the neurocognitive bases of shared predictions promises to illuminate much about the deep roots of human psychology and sociality.

## 7 Conclusion

This paper has explored the dual faces of human culture through the unifying lens of predictive coding. Viewing brains as prediction-optimizing systems provides insight into how both traditions and the arts aid cognitive adaptation in the social domain. We proposed complementary roles for cultural norms and creative imagination in training the predictive capacities of the social brain. The former propagate group-tested schemas, rituals, and practices that minimize collective uncertainty by providing efficient predictive frameworks for recurrent situations. Shared conventions reduce ambiguity about others’ likely behaviors and reactions during high-stake cooperative endeavors and routine social living.

Meanwhile, decoupled imaginary worlds of fiction, music, and make-believe facilitate the development of flexible social cognitive skills. The arts provide simulated play spaces to actively explore diverse perspectives, agents, and social dynamics well beyond those present in one’s immediate experience, while at the same time providing inputs that can be readily processed and exploited in specific real-life circumstances, but also turning out useful in future planning. For instance, dealing with counterfactual scenarios and unconventional narratives encountered in fictional worlds exercises the brain’s ability to contend with a wider array of mental models and outcomes which are essential for long-range executive tasks.

In this paper, we synthesized a scattered body of multidisciplinary evidence regarding the functions of traditions and the arts as part of an integrated evolutionary framework grounded in emerging predictive processing theories of cognition. This perspective reveals deep commonalities between the sciences of mind and culture. Both deal fundamentally with developing, propagating, and updating generative models of the world. If brains are essentially prediction engines, then culture constitutes the collective data sets and training environments for optimizing models of social worlds. Arts and traditions populate distinct but complementary niches in the cultural ecosystem, which play a key role in the acceleration of learning. The former specializes in imaginative exploration and expanding possibilities, while the latter conserves solutions and encodes wisdom into compressible rituals and norms. This framework integrates findings across anthropology, psychology, neuroscience, philosophy, and computational modeling into a common information-theoretic vocabulary elucidating what culture is, why it is so salient, and how it interacts with cognition. It provides an integrative foundation for future research examining cultural evolution through a predictive coding lens. A deeper understanding of our distinctive human cognition may emerge from examining its interdependence with the collective cultural-cognitive networks that shape its growth.

## Data availability statement

The original contributions presented in the study are included in the article/supplementary material, further inquiries can be directed to the corresponding author.

## Author contributions

AB: Investigation, Writing – original draft. AC: Investigation, Writing – original draft. AR: Investigation, Writing – original draft. PS: Conceptualization, Investigation, Supervision, Writing – original draft.
